# *Pastinaca sativa* L.: Nutritional Composition, Phytochemistry, and Pharmacological Properties Supporting Its Potential as a Functional Food and Therapeutic Agent

**DOI:** 10.1002/fsn3.71860

**Published:** 2026-05-07

**Authors:** Somanjana Khatua, Anish Nag, Surajit Sen, Jit Sarkar, Krishnendu Acharya, Daniela Calina, William N. Setzer, Javad Sharifi‐Rad

**Affiliations:** ^1^ Department of Botany, Faculty of Science University of Allahabad Prayagraj Uttar Pradesh India; ^2^ Department of Life Sciences CHRIST University Bangalore Karnataka India; ^3^ Department of Botany Fakir Chand College Kolkata India; ^4^ Molecular and Applied Mycology and Plant Pathology Laboratory, Centre of Advanced Study, Department of Botany University of Calcutta Kolkata West Bengal India; ^5^ Department of Clinical Pharmacy University of Medicine and Pharmacy of Craiova Craiova Romania; ^6^ Aromatic Plant Research Center Lehi Utah USA; ^7^ Department of Chemistry University of Alabama in Huntsville Huntsville Alabama USA; ^8^ Universidad Espíritu Santo Samborondón Ecuador; ^9^ Department of Medicine, College of Medicine Korea University Seoul Republic of Korea; ^10^ Centro de Estudios Tecnológicos y Universitarios del Golfo Veracruz Mexico

**Keywords:** *Pastinaca sativa* L., pharmacology, phytochemistry, proximate composition, safety assessment, traditional value

## Abstract

*Pastinaca sativa* L., commonly known as wild parsnip, is a biennial root vegetable (Apiaceae family), bearing a pale carrot‐like appearance. Historically cherished for its unique blend of spicy, nutty, and sweet flavor, this Eurasia‐originated plant has been widely utilized in culinary traditions including soup, cake, muffin, and pudding as well as in animal feed and even winemaking. Today, its significance extends far beyond gastronomy, as it is increasingly traded across Europe not only as a spice but also for its pharmaceutical and traditional medicinal uses, underscoring the growing interest. Research reveals that the herb is incredibly rich in starch, dietary fiber, vitamin C, and minerals while being low in sodium and calories. Beyond the nutritional merits, it harbors a wealth of bioactive components such as essential oils, terpenoids, polyphenols, flavonoids, polyacetylenes, coumarins, and furanocoumarins. As a result, a range of bioactivities, namely antioxidant, antibacterial, antifungal, cytotoxic, anti‐diabetic, and cardio‐protective effects, have been reported, arising interest for pharmacists. Such multifaceted bioactivity amplifies interest in *P. sativa* as a functional food with potential applications in preventing disease and health management. This review provides critical insights into the health‐promoting attributes of the plant, highlighting its promise as a valuable ingredient in modern diets and therapeutic formulations. Further in‐depth studies and clinical validations remain essential to unlock its full potential for real‐time applications.

## Graphical abstract

Food‐grade processing of *Pastinaca sativa* (parsnip) converts a nutrient‐rich plant matrix (dietary fiber, fructans, phenolics) through washing/slicing/enzymatic treatment and ingredient preparation into functional food ingredients, including fiber concentrates, antioxidant‐rich fractions, and parsnip powders/extracts. These outputs are linked to reported preclinical pharmacological activities, notably antioxidant, antimicrobial, antidiabetic (α‐amylase/α‐glucosidase inhibition), and cardioprotective effects.

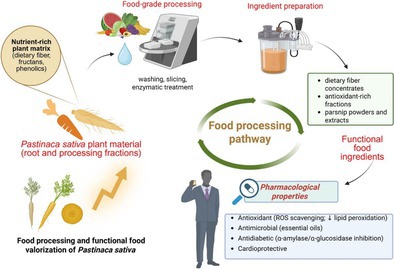

**Abbreviations**
AST: aspartate aminotransferase
BHA: butylated hydroxyanisole
BHT: butylated hydroxytoluene
DPPH: 2,2‐diphenyl‐1‐picrylhydrazyl
FRAP: ferric ion reducing antioxidant power
GC–MS: gas chromatography–mass spectrometry
HPLC: high‐performance liquid chromatography
IC_50_
: half‐maximal inhibitory concentration
MBC: minimum bactericidal concentration
MFC: minimum fungicidal concentration
MIC: minimum inhibitory concentration
RG: reduced glutathione
TBA: thiobarbituric acid

## 1 Introduction

Wild parsnip ( *Pastinaca sativa* L.), a monocarpic herbaceous species within the Apiaceae (Umbelliferae) family, is one of the barely explored species (Jianu et al. 2020). It originated possibly in Caucasus Mountains (a center for diversity of *Pastinaca* genus) and at present has spread throughout the world including United States and southern Canada due to domestication (Stelmach and Grzebelus 2023). However, the plant is sparsely distributed in India, Ceylon, and China (Nagaraju et al. 2021). The untamed variant is presently establishing itself in former fields, railroad embankments, roadside areas, and abandoned spaces (Arshad et al. 2025) (Contreras‐Pacheco et al. 2026). To date, two subspecies of the wild parsnip are found across Eurasia. *Pastinaca sativa* L. ssp. *sativa*, domesticated extensively in the Northern Hemisphere; ssp. *urens* [Req. ex Godron] Celak.; ssp. *sylvestris* [Mill.] Rouy (Jianu et al. 2020). Parsnip delights the palate with its sweet, nutty flavor and characteristic aroma. It produces small flowers and yellow fruits are typical reprehensive of the Mediterranean flora. Of particular fascination is its robust, funnel‐shaped taproot, ranging from pale white to vibrant yellow. This taproot is renowned as a versatile vegetable, enjoyed in its natural raw state, as well as when cooked, baked, fried, or roasted. Its most prevalent application is as an essential component in an array of soups, contributing both in dried form as a seasoning and in its fresh state. Additionally, it serves as a delightful inclusion in stews, salads, casseroles, pies, muffins, puddings, and various other culinary creations (Ušjak et al. 2022). It is also used as animal feed and in winemaking, while its fresh leaves and young buds are consumed as vegetables. Traditionally, parsnip has been valued for its nutritional and therapeutic properties, serving as an appetizer, digestive aid, and diuretic in various cultures. In addition, its seeds, which possess a bitter, dill‐like flavor, are used as spices and are traditionally believed to promote lactation in nursing mothers (Khadivi et al. 2023). The taproot is enriched with vitamins and minerals in comparison with carrots. Research has revealed that parsnip is particularly rich in vitamin C, minerals such as calcium and potassium and is a good source of dietary fibers (N. Č. Nikolić et al. 2014). Besides, it contains low amounts of sodium and fat resulting in low calorie making it suitable for individuals pursuing a healthy lifestyle (Tecucianu and Oancea 2019). However, parsnip, like other root vegetables, exhibits a firm texture that may limit its suitability for elderly consumers with mastication difficulties. Enzymatic treatment represents a promising approach to reduce tissue hardness, thereby enabling the development of texture‐modified parsnip products with improved palatability, preserved antioxidant capacity, and satisfactory technological properties (Kim et al. 2022). Consequently, parsnips contain a broad range of bioactive components, including phenolic compounds and flavonoids, which contribute to their value as a source of functional nutrients, particularly in the context of elderly nutrition. To meet consumer demand, the plant is widely cultivated as a biennial crop, and different parts are commercially traded in Europe for use as spices, pharmaceutical preparations, and in traditional medicine.

(Stelmach and Grzebelus 2023; Tosun et al. 2019). However, the plant is generally considered a nuisance being associated with photo‐sensitization of livestock and humans (Stegelmeier et al. 2019). However, the cultivated variant harbors diminished levels of the problematic furanocoumarins compared to its wild counterpart (Averill and DiTommaso 2007). In recent years, *P. sativa* has thus attracted growing scientific interest owing to its untapped pharmacological potential, rich phytochemical profile, and relevance in sustainable food systems (Khadivi et al. 2023). This surge aligns with broader trends in the last 5 years, which highlight a marked shift towards the development of natural, plant‐based functional foods, bioactive‐rich dietary supplements, and clinical evaluations of traditional botanicals for chronic disease prevention and management (Tachie et al. 2023). As global consumers increasingly seek foods with added health benefits, *P. sativa* stands out as a promising yet underutilized candidate. This comprehensive review consolidates current knowledge on the nutritional composition, bioactive constituents, and pharmacological properties of the plant, offering new insights into its utility in modern medicine, functional food innovation, and nutraceutical development.

## 2 Review Methodology

A comprehensive search for peer‐reviewed articles was conducted using leading scientific databases including ScienceDirect, Web of Science, Scopus, American Chemical Society, Google Scholar, PubMed/Medline, and Wiley. The search utilized specific keywords and phrases such as “wild parsnip,” “ *Pastinaca sativa* ,” “ *Pastinaca sativa* antioxidant,” “ *Pastinaca sativa* phytochemical,” and “ *Pastinaca sativa* description,” combined with Boolean operators “AND,” “OR,” and “NOT” to refine and broaden the scope of the literature search. This methodology aimed to capture a wide range of research articles, review papers, and editorials pertinent to the study of *Pastinaca sativa* . Inclusion Criteria: articles published from 2000 onwards were included to ensure the incorporation of the most recent and relevant scientific findings. Articles published exclusively in English were considered to avoid translation‐related inaccuracies and to maintain a consistent quality of the review. The review focused on peer‐reviewed research articles, comprehensive review articles, and high‐quality editorials that provide substantial insights into the botany, phytochemistry, nutritional value, and pharmacological properties of *Pastinaca sativa* . All two‐dimensional structures of phytochemicals and relevant information were obtained from the PubChem database (https://pubchem.ncbi.nlm.nih.gov/). Studies with a primary focus on *Pastinaca sativa* and its related species, addressing aspects such as biochemical composition, health benefits, and traditional uses, were prioritized. Exclusion Criteria: articles published prior to 2000 were excluded to prevent the inclusion of outdated information. Non‐English articles were excluded due to the risk of translation inaccuracies and to ensure uniformity in the review process. Non‐peer‐reviewed sources, including conference abstracts, book chapters, and gray literature, were excluded to uphold the quality and reliability of the review. Studies that did not directly address *Pastinaca sativa* or were only tangentially related were excluded. Articles lacking methodological rigor, clear experimental design, or conclusive results were excluded to ensure the robustness of the scientific evidence presented. The references were chosen for their innovativeness, clarity of explanation, systematic approach, and high impact, thereby providing a comprehensive and up‐to‐date understanding of *Pastinaca sativa* .

## 3 Botanical Description and Geographical Distribution

**FIGURE 1:**
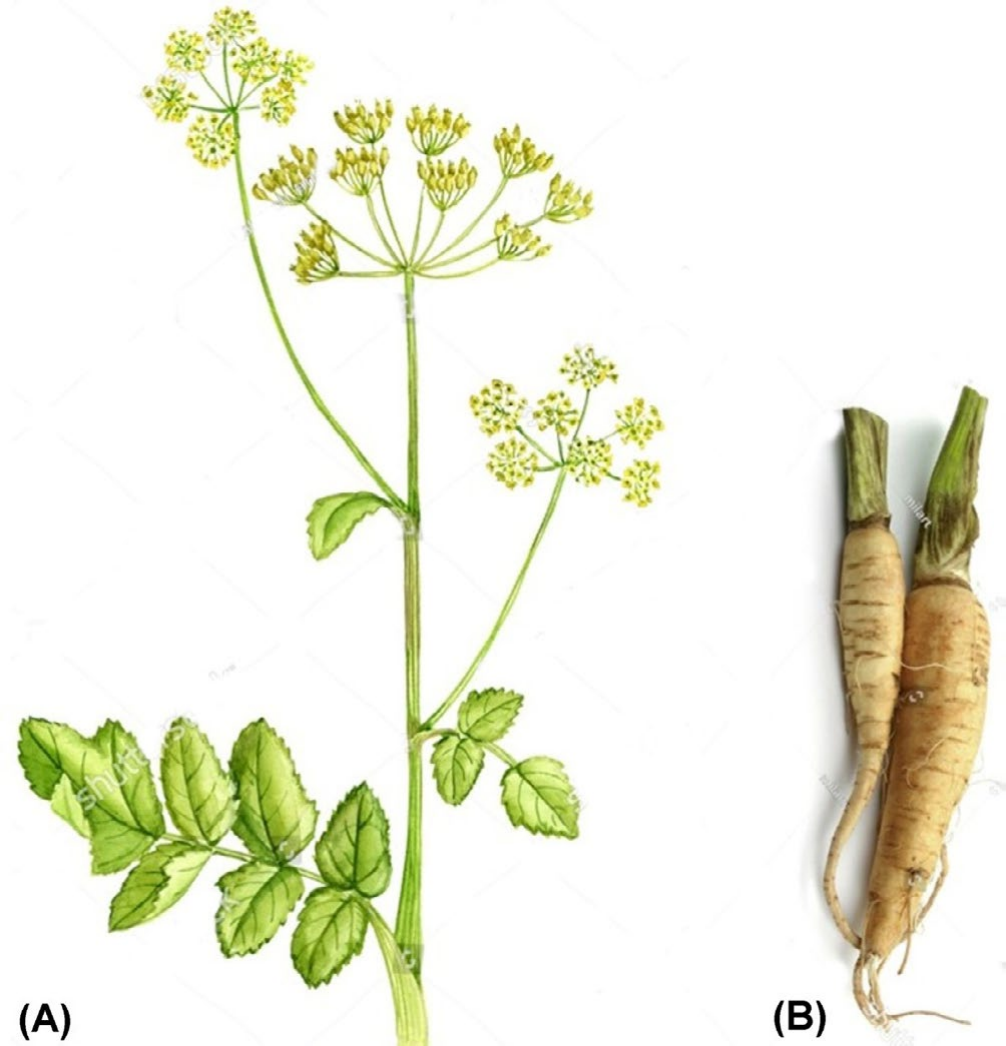
*Pastinaca sativa* . (A) Flowering stem (B) taproot.

**FIGURE 2:**
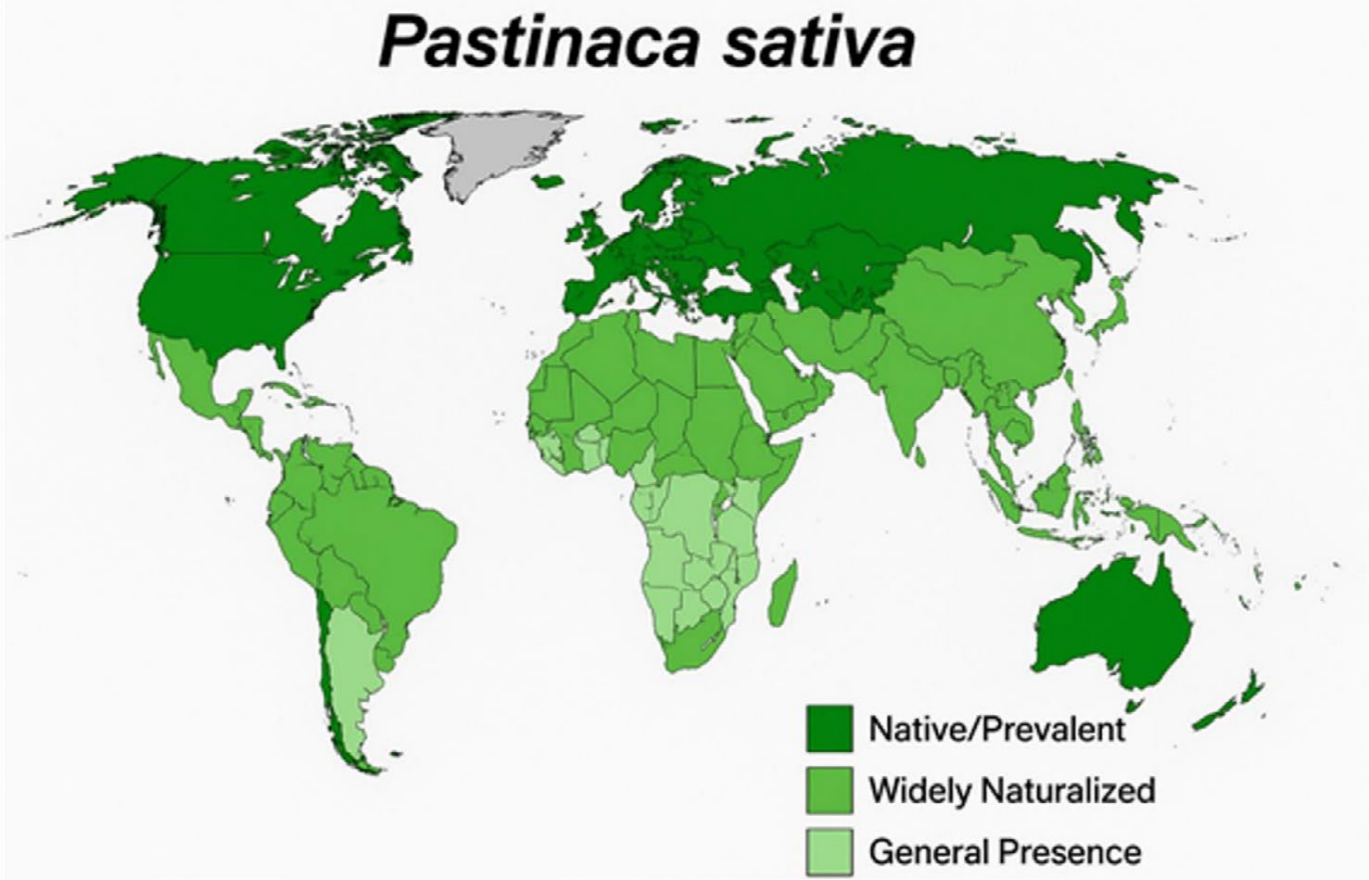
Geographical distribution of *Pastinaca sativa* .

The name *Pastinaca* is thought to be derived from the Latin word pastino which indicates “preparing the land for planting of the vine”, but might alternatively arise from the Latin word pastus, which means “*food*” signifies the edible root (Fernald 1950; Menemen and Jury 2001). The common names of *Pastinaca* are: parsnip, bird's nest, hart's‐eye, heeltrot, hockweed, madnip, queen weed, and tank (Jaques 1959; Klaber 1942). *Pastinaca sativa* has many common names in various languages such as zardak and wild carrot in Persian, parsnip in English, cujtive and panipainais in French, jazar in Arabic, and kajer in Hindi (Crellin and Philpott 2010). While typically a biennial, the plant has the capacity to exhibit characteristics akin to those of a monocarpic perennial. It belongs to the carrot family, Apiaceae (Baskin and Baskin 1979; Sullivan 2004; Zomlefer 1994). The tall (0.3–1 m), robust, herbaceous wild parsnip has a long, thick, and deep taproot. It boasts alternate, pinnately compound leaves with a soft and feathery appearance, measuring around 15 cm in length. The plant undergoes a maturation process spanning over 2 years, culminating in the development of a grooved aerial shoot and the blossoming of its flowers. The petioles of lower leaves are longer than those of upper leaves, which can also be sessile. Each leaf contains 5–15, oblong to ovate, lobed, or serrated leaflets that are 5–10 cm in long (Sullivan 2004). Inflorescences are large, compound umbels, approximately 10–20 cm wide more or less flat‐topped, with 6–25 straight rays supporting the umbellets (Figure 1). The primary (terminal) umbel bears bisexual flowers towards the periphery; while the staminate flowers are arranged towards the center. The secondary (axillary lateral) umbels encompass a decreasing number of bisexual flowers often only with staminate flowers in the highest umbel orders. The bisexual flowers exhibit a protandrous nature, progressing first through a staminate stage and subsequently transitioning into a pistillate phase. Pedicels are long or longer than fruits; petals 5, entire, involute, yellow, usually ebracteate or ebracteolate; sepals are minute or absent. Fruits are schizocarpic, glabrous, elliptic to obovate, low‐ribbed and 5–7 mm in length (Lorenzi and Jeffery 1987; Sullivan 2004). Fruits of the lateral umbels are smaller than that of terminal umbels. Fruits contain two mericarps, each with one seed; ovary inferior. Sixteen species of *Pastinaca* have been identified worldwide, according to the World Flora Online (WFO) database, and *P. sativa* has been divided into two subspecies (ssp. *sativa* and ssp. *urens*) (Table 1). Many contrasting characters distinguish among the species under the genus *Pastinaca* (Table 1). In the realm of *P. lucida* , its leaves exhibit simplicity and a glabrous texture on both upper and lower surfaces, setting it distinctly apart from other members of the *Pastinaca* genus. Unlike its counterparts, which feature pinnate leaves adorned with hairs on both sides, *P. lucida* boasts this unique characteristic. Divergence also extends to various attributes such as leaf shape, size, presence or absence of bracts and bracteoles, petal color and hairiness, as well as ray length and quantity, along with the presence or absence of brown veins. *Pastinaca sativa* , in contrast, bears brown veins, glabrous or slightly covered yellow petals, leaves that are once‐pinnate and covered in hair on both surfaces, and is characterized by its ebracteate or ebracteolate nature. Furthermore, differentiation within *P. sativa* ssp. *sativa* is attainable through meticulous examination of the stem and the presence of leaf hairs (Averill and DiTommaso 2007). The plant is prevalent throughout temperate Europe, ranging from Eastern Europe into Western Central Asia, via Turkey into Iran and the Caucasus, and south‐eastward from the Pamirs to the western Himalayan range. It is also found in Africa, South America, New Zealand, and Australia (Figure 2) (Holm et al. 1979; Hultén and Fries 1986; USDA‐ARS 2008). The species is widely naturalized in many countries including China, Japan, Belgium, France, Germany, Italy, Netherlands, Spain, Ukraine, UK, Canada, and the USA (USDA‐ARS 2008). In North America, *P. sativa* is mainly present in eastern regions, but it is widely naturalized across the United States. It is distributed in all American states Except Hawaii, Mississippi, Alabama, Georgia, and Florida. It is also reported that in Canada, parsnip occurs in all provinces and territories except Nunavut (Darbyshire and James 2003). It has been found that alkaline, and moist soils are the best suitable parameter for growth of the plant; however the vegetable can survive under poor soil condition also (Averill and DiTommaso 2007).

**TABLE 1:** Contrasting characters of 16 species of *Pastinaca* (Averill and DiTommaso 2007; Menemen and Jury 2001).

Species	Contrasting characters		
	Leaves	Petals	Bracts and bracteoles
*P. lucida* L.	Simple and glabrous on both sides	Pinkish	Absent
*P. zozimoides* Fenzl	Pinnate and hairy on both sides		
*P. hirsuta* Pančić	Pinnate and hairy on both sides	Yellow, glabrous or subglabrous on dorsal surface	Present
*P. pimpinellifolia* M. Bieb.	Pinnate and hairy on both sides, elliptic to ovate‐triangular, mericarp elliptic to ovate, rays generally more than 10	Yellow, hairy	Present
*P. armena* Fisch. & C.A. Mey.	Pinnate and hairy on both sides, oblong to elliptic, mericarp elliptic to orbicular, rays generally less than 10	Yellow, hairy	Present
*P. sativa* L.	1‐pinnate, hairy on both sides, rays equal or subequal, brown veins present	Yellow, glabrous or subglabrous, mericarp elliptic, commissural surface glabrous, very rarely waxy	Absent
*P. argyrophylla* Delip.	Pinnate, often glabrous, with serrated or pinnatifid pinnae.	Yellow, ovate petals in terminal and lateral umbels	Present
*P. aurantiaca* (Albov) Kolak.	Pinnate, typically glabrous, and the pinnae are serrate or pinnatifid	Yellow and ovate	Absent
*P. clausii* (Ledeb.) Calest.	Pinnate, with obovate terminal segments.	Small, yellow, and arranged in umbels	Absent
*P. erzincanensis* Menemen & Kandemir	2–3 pinnate or ternate with furcately divaricated segments.	White and ovate to obovate	Present
*P. gelendostensis* (Yild. & B. Selvi) Hand	Pinnate and glabrous on both sides	Yellow, glabrous	Absent
*P. glandulosa* Boiss. & Hausskn.	Pinnate and glabrous on both sides	Small, yellow, and arranged in compound umbels	Absent
*P. kochii* Duby	1–2 pinnately compound, leaflet oblong, coarsely toothed	Small, yellow, and arranged in flat‐topped umbels	Present
*P. trysia* Stapf & Wettst.	Pinnate, glabrous on both surfaces, with serrate or pinnatifid leaflets that are sessile	Petals ovate, yellow, and incurved at the apex	Absent
*P. vanensis* Demir, Sefalı & Yapar	Pinnate and hairy on both sides	White‐dirty to white petals	Absent
*P. yildizii* Dirmenci	1‐ pinnate, oblong in outline, leaflets 6–8 paired, broadly ovate to oblong	Purple, equal, hairy	Present

## 4 Ethnopharmacology

Since Roman times, parsnip has been domesticated owing to its long, fleshy, and edible root (Kenari et al. 2021). The naturally sweet flavor of *P. sativa* contributed to its widespread popularity in Europe prior to the introduction of refined sugar. Currently, parsnip is widely used for culinary purposes in the United Kingdom and Ireland, where it is consumed raw or prepared by boiling, roasting, baking, steaming, frying, or cooking, owing to its characteristic flavor and high dietary fiber content. It is used in the preparation of purées, jams, and marmalades, incorporated into stews, soups, salads, pies, puddings, cakes, and casseroles, and is also dried for use as a seasoning or processed for canning (Rawson et al. 2010). During lent, the vegetable is taken with salt fish and boiled egg. Roasted parsnip is regarded as an essential dietary item in Christmas dinner and enjoyed in customary Sunday roast (Lim 2015). In Scotland, *P. sativa* is praised as a staple herbal in the regular diet and subjected to a variety of preparations. The vegetable is also deliberated as a root aphrodisiac in combination with celery (Augustin and Butnariu 2022). Making beer and wine using parsnip is also a tradition in Britain and Ireland. In Ireland, cottagers boil the roots in water and hops followed by fermentation that eventually produce a pleasant beer (Lim 2015). Parsnip is also used as an ingredient in infant food and to feed livestock (Akbarmivehie and Baghaei 2016; Tosun et al. 2019). Apart from the roots, leaves and young shoots are cooked as well with other greens and included in soups (Ušjak et al. 2022). The seed is considered bitter aromatics that increases milk yield. It is used as a condiment and has similar taste to dill (an aromatic annual herb of the parsley family) (Lim 2015; Tosun et al. 2019). Additionally, different parts of parsnips have been used for medicinal purposes by the Romans and Greeks in ancient times (Kalloo 1993). For instance, in Serbia, the infusion crafted from the roots, leaves, or fruits garner acclaim for its advantageous effects on digestion and diuresis, as well as its ability to enhance appetite and stimulate milk production. Similarly, in Italy, infusions derived from the roots or leaves serve as a valued remedy for promoting bile secretion, supporting dietary goals, and fostering diuresis (Ušjak et al. 2022). Tosun et al. (Tosun et al. 2019) reported that a whole plant could be applied to increase appetite, better digestion, and as a diuretic (Table 2). In Iranian traditional remedy, parsnip is renowned as a stomach astringent, liver and uterine tonic, and ovulation stimulator. Indeed, it is entitled as semen or sexual desire increaser and fertility agent (Hakimi et al. 2020).

**TABLE 2:** Ethnopharmacological uses of *Pastinaca sativa* (Parsnip).

Usage category	Details	References
Historical use	Domesticated since Roman times for its edible root.	(Kenari et al. 2021)
Culinary uses	Widely used in the UK and Ireland for its sweet flavor and high dietary fiber. It can be consumed raw, cooked in various methods, and is a traditional component of Christmas dinner and Sunday roast.	(Rawson et al. 2010) (Lim 2015)
Beverage production	Traditionally used to make beer and wine in Britain and Ireland.	(Lim 2015)
Infant food	Ingredient in infant food and used to feed livestock.	(Akbarmivehie and Baghaei 2016; Tosun et al. 2019).
Leaf and shoot use	Cooked with other greens and included in soups.	(Ušjak et al. 2022)
Seed use	Seeds are bitter aromatics that increase milk yield and are used as a condiment similar to dill.	(Lim 2015; Tosun et al. 2019)
Medicinal uses	Used by ancient Romans and Greeks for digestion, diuresis, appetite stimulation, and milk production. In Serbia, infusions from roots, leaves, or fruits are used to enhance digestion and diuresis.	(Kalloo 1993) (Ušjak et al. 2022)
Traditional medicine	In Iranian traditional medicine, parsnip is used as a stomach astringent, liver and uterine tonic, ovulation stimulator, and fertility agent.	(Hakimi et al. 2020)

## 5 Phytochemistry

**FIGURE 3:**
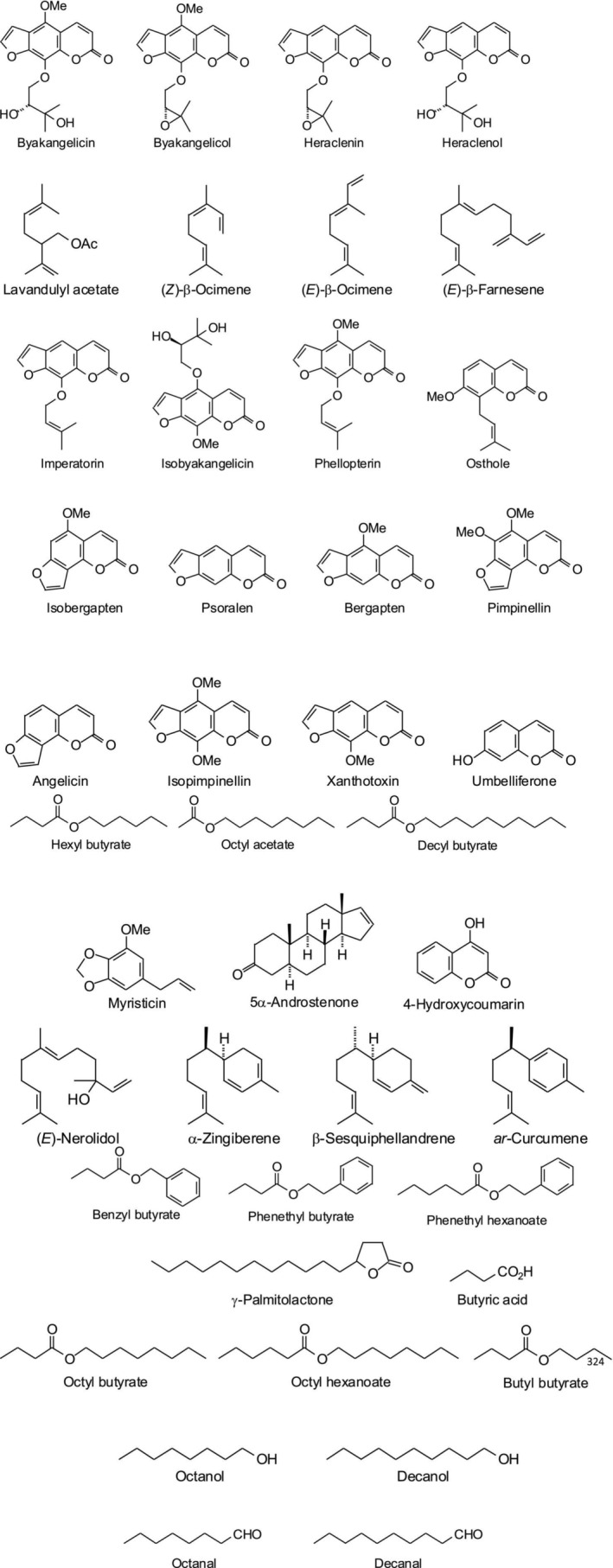
Two‐dimensional structures of major phytochemicals isolated from *Pastinaca sativa* (https://pubchem.ncbi.nlm.nih.gov/).

The plant *P. sativa* is rich in various phytocompounds. A large number of phytochemical classes such as essential oils, terpenoids, polyphenols, flavonoids, polyacetylenes, coumarins, and furanocoumarins were isolated from different parts of the plant (Chizzola 2010; Emami et al. 2010; Fahmy et al. 1956; Jamshidi et al. 2020; Janeczko and Skoczowski 2005; Kurkcuoglu et al. 2006; Kviesis et al. 2019; Madari and Jacobs 2004; Matejić et al. 2014; Mongeau et al. 1994; Nemych et al. 2018; Soine et al. 1956). The major compounds such as isobyakangelicin, byakangelicol, heraclenol, heraclenin, isobergapten, isobergapten, and byakangelicin were isolated from *P. sativa* . Furanocoumarin is the dominant class of compounds in *P. sativa* , and reported compounds were imperatorin, pimpinellin, isopimpinellin, bergapten, phellopterin and methoxsalen (Amri 2014; Harper et al. 2015; Heywood 2014; Jensen and Hansen 1939; Kenari et al. 2021; Picardo et al. 1986; Seavy and Rizzolo 2013; Terreaux et al. 2003; Walling and Walling 2018). Many minerals such as potassium, manganese, magnesium, phosphorus, zinc, iron, and vitamins like B1, B2, C, E, and K were reported in the root. The aerial part and root of the plant also contain other compounds such as polysaccharides, organic acids, hydroxycinnamic acids, hydroxycinnamic acids and amino acids. The phytochemicals of *P. sativa* are listed in Table 3 and Figure 3.

**TABLE 3:** List of phytocompounds isolated from *Pastinaca sativa* (Kenari et al. 2021).

S/N	Name of the compounds	Phytochemical class/source	Structural formula	Molecular weight (g/mol)	Isolated from
1	Lavandulyl acetate	Ester	C_12_H_20_O_2_	196.29	Aerial part
2	γ‐Palmitolactone	Lactone	C_16_H_30_O_2_	254.41	
3	Angelicin	Coumarin	C_11_H_6_O_3_	186.16	Root/Aerial part
4	Bergapten		C_12_H_8_O_4_	216.19	
5	Xanthotoxin		C_12_H_8_O_4_	216.19	
6	Imperatorin		C_16_H_14_O_4_	270.28	Root
7	Psoralen		C_11_H_6_O_3_	186.16	Root/Aerial part/Seed
8	Octyl butyrate	Fatty acid ester	C_12_H_24_O_2_	200.32	Root
9	Myristicin	Phenylpropene	C_11_H_12_O_3_	192.21	
10	(*E*)‐β‐Ocimene	Monoterpene	C_10_H_16_	136.23	Root/Aerial part
11	(*E*)‐β‐Farnesene	Sesquiterpene	C_15_H_24_	204.35	
13	Octyl hexanoate	Fatty acid ester	C_14_H_28_O_2_	228.37	Root
14	α‐Terpinolene	Monoterpene	C_10_H_16_	136.23	Root/Aerial part
15	5α –Androstenone	Steroid	C_19_H_28_O	272.4	Root
16	Bergapten	Coumarin	C_12_H_8_O_4_	216.19	Root/Aerial part
17	Pimpinellin		C_13_H_10_O_5_	246.21	Seed Seed
18	Methoxsalen		C_12_H_8_O_4_	216.19	
19	Isopimpinellin		C_13_H_10_O_5_	246.21	
21	Phellopterin		C_17_H_16_O_5_	300.3	
22	Byakangelicol		C_17_H_16_O_6_	316.3	
23	Heraclenin	Coumarin	C_16_H_14_O_5_	286.28	
24	Isobergapten		C_12_H_8_O_4_	216.19	
25	Byakangelicin		C_17_H_18_O_7_	334.3	
26	Heraclenol		C_16_H_16_O_6_	304.29	
27	Isobyakangelicin		C_17_H_18_O_7_	334.3	
29	Myristicin	Phenylpropanoid	C_11_H_12_O_3_	192.21	Seed/root
30	Octyl hexanoate	Fatty acid ester	C_6_H_11_O_2_^−^	115.15	Root
31	Butyl butyrate	Ester	C_8_H_16_O_2_	144.21	Seed
32	(Z)‐b‐Ocimene	Monoterpene	C_10_H_16_	136.23	Seed/Aerial part
33	Octanal	Aldehyde	C_8_H_16_O	128.21	Seed Seed
34	Hexyl butyrate	Ester	C_10_H_20_O_2_	172.26	
35	Octyl acetate	Ester	C_10_H_20_O_2_	172.26	
36	Decanal	Aldehyde	C_10_H_20_O	156.26	
37	Octanol	Alcohol	C_8_H_18_O	130.229	
38	α‐Zingiberene	Sesquiterpene	C_15_H_24_	204.35	
39	Decanol	Alcohol	C_10_H_22_O	158.28	
40	β‐Sesquiphellandrene	Sesquiterpene	C_15_H_24_	204.35	
41	*ar*‐Curcumene		C_15_H_22_	202.33	
42	Decyl butyrate	Ester	C_14_H_28_O_2_	228.37	
43	Benzyl butyrate	Ester	C_11_H_14_O_2_	178.23	
44	Phenylethyl butyrate	Ester	C_12_H_16_O_2_	192.25	
45	(*E*)‐Nerolidol	Sesquiterpene alcohol	C_15_H_26_O	222.37	
46	Phenylethyl hexanoate	Ester	C_14_H_20_O_2_	220.31	
47	Butyric acid	Fatty acid	C_4_H_8_O_2_	88.11	
48	Osthol	Coumarin	C_15_H_16_O_3_	244.28	
49	Umbelliferone		C_9_H_6_O_3_	162.14	
50	4‐Hydroxycoumarin		C_9_H_6_O_3_	162.14	

Recent studies have also clarified the principal methods used for the extraction and characterization of phytochemicals in *P. sativa* (Di Girolamo et al. 2025). Volatile constituents, particularly essential‐oil esters and terpenoids, have been isolated by hydrodistillation and characterized mainly by gas chromatography–mass spectrometry (GC–MS), together with retention index analysis, enabling the identification of compounds such as octyl acetate, octyl butanoate, and hexyl butanoate (Di Girolamo et al. 2025; Eruygur et al. 2024). For non‐volatile fractions, solvent extraction with ethanol or hexane has been used to obtain leaf and fruit extracts enriched in phenolic and flavonoid constituents, which were subsequently assessed using spectrophotometric assays for total phenolic and flavonoid content. In parallel, recent analytical work in Apiaceae has highlighted the value of validated ultra‐high‐performance liquid chromatography with photodiode array detection (UHPLC‐PDA), high‐performance thin‐layer chromatography (HPTLC), and tandem mass spectrometric approaches for the quantitative characterization of phenolics, flavonoids, and coumarins. Collectively, these methodological advances provide a stronger analytical basis for the identification and comparative evaluation of key phytochemicals in *P. sativa* (Kureshi et al. 2025).

## 6 Pharmacological Properties

**FIGURE 4:**
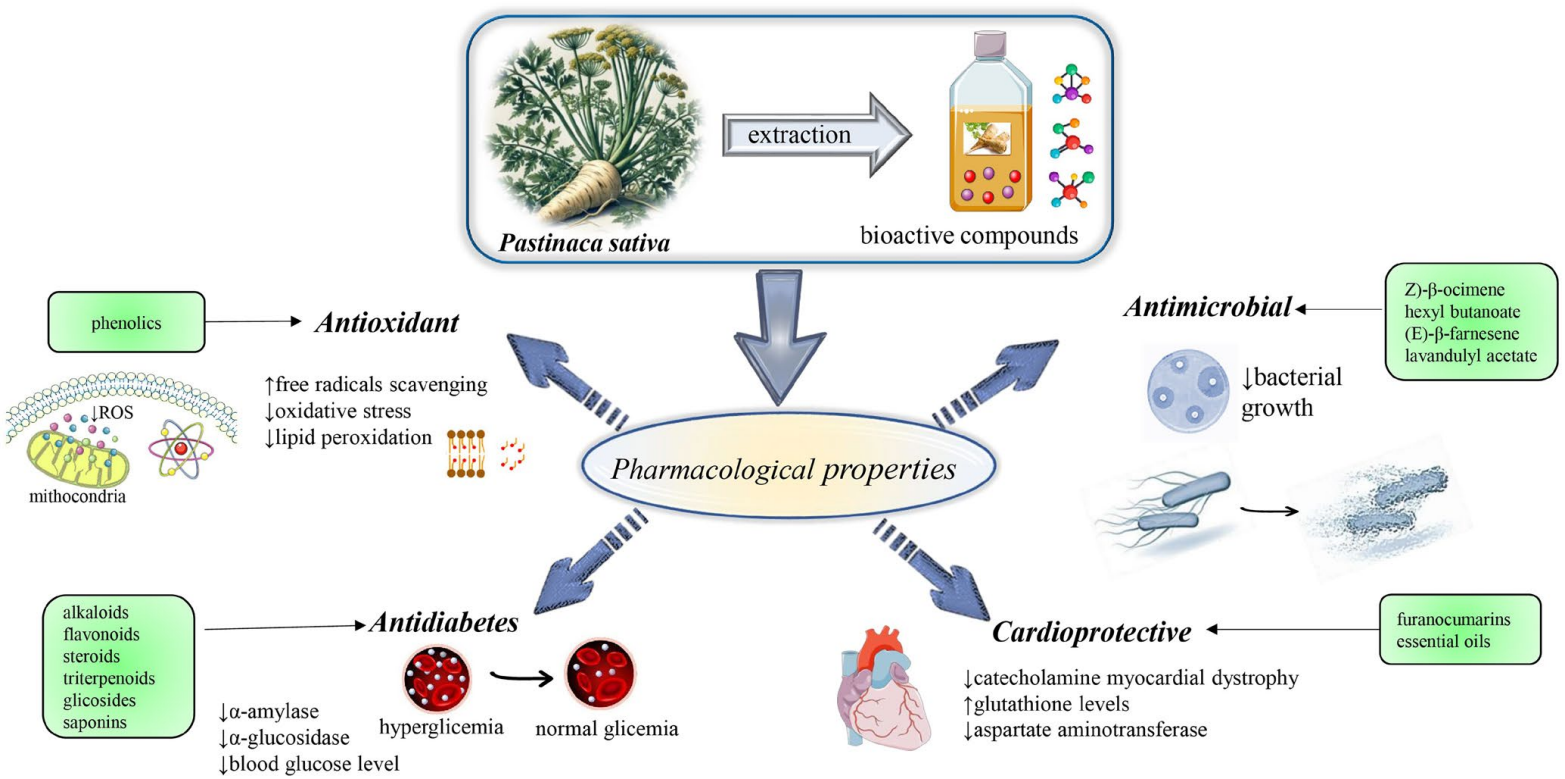
Summarized scheme regarding pharmacological properties of *Pastinaca sativa* . Phenolic compounds contribute to antioxidant activity by enhancing free radical scavenging and reducing reactive oxygen species (ROS), oxidative stress, and lipid peroxidation. Antimicrobial activity has been associated with volatile constituents such as (Z)‐β‐ocimene, hexyl butanoate, (E)‐β‐farnesene, and lavandulyl acetate, which inhibit microbial growth. Antidiabetic effects have been linked to alkaloids, flavonoids, steroids, triterpenoids, glycosides, and saponins, which reduce α‐amylase and α‐glucosidase activities and thereby lower blood glucose levels, contributing to normoglycemia. Cardioprotective effects have been related to furanocoumarins and essential oils, which may attenuate catecholamine‐induced myocardial dystrophy, increase glutathione levels, and decrease aspartate aminotransferase, thereby helping to protect myocardial tissue from oxidative damage.

*Pastinaca sativa* harbors a diverse array of advantageous properties that contribute positively to an extensive spectrum of health conditions. These encompass conditions such as cholecystitis, constipation, anorexia, abdominal discomfort, bladder atony, spastic enterocolitis, mild insomnia, nephritis, dysuria, renal colic, endocrine imbalances (including menstrual syndrome), rheumatism, avitaminosis, obesity, vascular ailments, infections, diminished appetite, dysmenorrhea, fever, atherosclerosis, detoxification, anemia, and diabetes (Augustin and Butnariu 2022). Nevertheless, the documentation of medicinal benefits of the plant remains relatively sparse in the existing literature (Table 4; Figure 4).

**TABLE 4:** Pharmacological activities of different parts extracts of the *Pastinaca sativa* .

Pharmacological activity	Tested plant parts	Type of study in vitro/in vivo	IC_50_ value/doses	Effects/mechanisms	References
Antioxidant	Seed (essential oil)	In vitro	Dose: 100–300 mg/mL	Inhibited primary oxidation of sunflower oil	(Jianu et al. 2020)
	Fruit and above ground parts (essential oil)	In vitro	Dose: 50–250 ppm	Radical scavenging	(Tosun et al. 2019)
	Powder, ethanol extract and aqueous extract	In vitro	Dose: 0.3%–0.4% for powder; 0.25%–0.35% for the extracts	Inhibited lipid oxidation in beef burgers	(Akbarmivehie and Baghaei 2016)
	Root (80% ethanol extract)	In vitro	IC_50_: 1.59–2.49 mg/mL	Radical scavenging	(N. Č. Nikolić et al. 2014)
	Roots and tubers (methanol extract)	In vitro	Not mentioned	Reduction of Fe^3+^ to Fe^2+^	(Halvorsen et al. 2002)
Antioxidant and anti‐cytolytic	Thick extract	In vivo	Dose: 100 and 200,100 mg/kg	↑↑RG ↓↓AST, TBA	(N. Symonenko et al. 2022)
Antimicrobial	Root, leaf, stem, flower, and ripe fruit (essential oil)	In vitro	MIC: 1–8 mg/mL; MBC: 2–16 mg/mL; MFC: 0.5–8 mg/mL	Not investigated	(Ušjak et al. 2022)
	Aerial parts (essential oil)	In vitro	MIC and MBC: 0.72–92.5 mg/mL; MFC: 46.2 mg/mL	Not investigated	(Matejić et al. 2014)
	Root (essential oil)	In vitro	MIC: 1.25–2.5 mg/mL; MFC: 1.25–5 mg/mL	Not investigated	(M. Nikolić et al. 2015)
Anti‐diabetic	Entire plant (methanol extract)	In vitro and in vivo	IC_50_: 91.69 and 88.05 μg/mL for α‐amylase and α‐glucosidase	Blood glucose level in diabetic rats	(Nagaraju et al. 2021)
Cardioprotective	A tablet form of a thick extract	In vivo	Dose: 100 and 200 mg/kg	AST, TBA, serum urea content	(N. A. Symonenko et al. 2021)

Abbreviations: ↑, increase; →, conversion; ↓, decrease; AST, aspartate aminotransferase; Fe2+, ferrous ion; Fe3+, ferric ion; IC_50_, half‐maximal inhibitory concentration; MBC, minimum bactericidal concentration; MFC, minimum fungicidal concentration; MIC, minimum inhibitory concentration; RG, reduced glutathione; TBA, thiobarbituric acid.

### 6.1 Antioxidant Activity

Oxidative stress, a condition characterized by an imbalance between free radicals and antioxidants in the body, plays a fundamental role in the pathophysiology of chronic diseases, highlighting the importance of antioxidants in mitigating the damage caused by oxidative stress and potentially preventing disease progression (Chaudhary et al. 2023). *P. sativa* has been extensively studied for its potent antioxidant properties, demonstrating significant free radical scavenging activity and the ability to mitigate oxidative stress, making it a promising natural source for combating oxidative damage (Jianu et al. 2020). In a study, parsnip smooth roots varied in weight and were bought from the market and further 80% ethanol extract was prepared. The highest phenolic content was found in the extract prepared from the lowest weight of root. As a result, the fraction exhibited the strongest DPPH^•^ scavenging effect with EC_50_ of 1.59 mg/mL. HPLC analysis indicated the presence of various phenolics in all the preparations where a positive correlation was found between kaemferol glucoside and antioxidant efficacy (N. Č. Nikolić et al. 2014). In another research work, methanol extract was prepared from a range of dietary plants revealing that carrots exhibited the lowest antioxidant content among all the roots and tubers analyzed (i.e., 0.04 mmol/100 g), a value approximately half that of parsnip (Halvorsen et al. 2002). In a recent experimentation, dried seeds of *P. sativa ssp. sylvestris* were used to obtain the essential oil by the method of steam distillation. GC–MS analysis portrayed presence of at least 32 components in the oil where octyl acetate was recorded as the main component. Moreover, the isolated essential oil exhibited an excellent potential for inhibition of primary and secondary oxidation products for cold‐pressed sunflower oil where the outcome was somewhat equivalent with standards, namely BHA and BHT. The antioxidative property was further validated through β‐carotene‐linoleic acid bleaching and DPPH^•^ quenching assays (Jianu et al. 2020). The same radical scavenging method has also been used to understand antioxidant activities of methanol extracts isolated from *P. sativa* ssp. *urens*. Results showed better antioxidant activity from the above‐ground parts than that of the fruit extracts. At the level of 50 ppm, DPPH^•^ scavenging activity of fruits and above‐ground parts were 3.28% and 23.1% respectively. This could be justified by the presence of phenolic compounds in higher amount in the preparation of above‐ground parts (Tosun et al. 2019). Interestingly, parsnip powder, ethanol extracts, and aqueous extracts of parsnip are very effective against lipid oxidation and have potential as natural antioxidants in beef burgers (Akbarmivehie and Baghaei 2016). Overall, the antioxidant evidence for *P. sativa* is encouraging; however, direct comparison across studies remains difficult because of substantial variation in plant part, extraction solvent, assay system, and dose range. Most available studies rely on chemical antioxidant assays or food‐model systems rather than biologically relevant in vivo or clinical endpoints. These limitations indicate the need for standardized phytochemical characterization and more physiologically relevant models to clarify the true nutritional and functional significance of the reported antioxidant effects.

### 6.2 Antimicrobial and Antifungal Activity

Natural compounds, such as essential oils and plant extracts, have been extensively studied for their antimicrobial properties, showing efficacy against a wide range of pathogenic microbes, including bacteria, fungi, and viruses (Alshehri et al. 2022; Kumari et al. 2024; Rybczyńska‐Tkaczyk et al. 2023). These bioactive compounds often target microbial cell membranes, disrupt metabolic pathways, and inhibit enzyme activity, making them potent agents in the fight against microbial infections and resistance (Kumari et al. 2024). *P. sativa*, is recognized not only for its culinary and nutritional value but also for its antimicrobial properties (Kenari et al. 2021). Among these, its antimicrobial activity stands out, with various studies highlighting the plant's potential in inhibiting the growth of pathogenic microorganisms. The essential oil extracted from the aerial parts of *P. sativa* has demonstrated significant antimicrobial effects, attributable to its rich composition of compounds such as (*Z*)‐β‐ocimene, hexyl butanoate, (*E*)‐β‐farnesene, and lavandulyl acetate (Hochma et al. 2021). To elucidate antimicrobial effect, essential oil was isolated from the aerial parts of *P. sativa* by hydro‐distillation technique. Chemical analysis portrayed presence of (*Z*)‐β‐ocimene and hexyl butanoate as the chief components, while (*E*)‐β‐farnesene and lavandulyl acetate were also detected in appreciable quantity. Further investigation revealed *Bacillus cereus* ATCC 10876 as the most susceptible bacterium to the oil under study exhibiting the lowest MIC of 0.72 mg/mL. In addition, *Escherichia coli* ATCC 25922, *Pseudomonas aeruginosa* ATCC 9027, *Staphylococcus aureus* ATCC 25923 exhibited susceptibilities as well with MIC values ranging from 23.1 to 46.2 mg/mL. However, *Salmonella enteritidis* ATCC 13076 and *Listeria monocytogenes* ATCC 15313 were resistant each showing MIC of 92.5 mg/mL. Nevertheless, the oil was able to inhibit growth of *Candida albicans* ATCC 10231 with MFC of 46.2 mg/mL suggesting that *P. sativa* could be regarded as a nature‐derived antimicrobial and antifungal agent (Matejić et al. 2014). In another study, essential oil was extracted from roots, leaves, stems, flowers, and ripe fruits of cultivated *P. sativa* ssp. *sativa*. GC–MS analysis exhibited the dominant presence of myricetin. Consequently, the oil portrayed potent antibacterial activity evident by growth inhibitory effect against *Staphylococcus aureus* , *Bacillus cereus* , *Listeria monocytogenes* , *Escherichia coli* , *Salmonella typhimurium* , and *Enterobacter cloacaea* where MIC values varied from 1 to 4 mg/mL. Besides, promising anticandidal effect against *Candida tropicalis* , *C. parapsilosis* , *C. krusei* , *C. glabrata* , and *C. albicans* where MFC values ranged from 0.25 to 1 mg/mL (Ušjak et al. 2022). Similar trend of anticandidal activity of essential oil from *P. sativa* has also been reported by (N. Č. Nikolić et al. 2014). Taken together, the antimicrobial findings suggest promising activity of *P. sativa* essential oils, but the reported potency varies markedly across studies. This inconsistency is likely related to differences in subspecies, plant organ, extraction method, and volatile composition. In addition, the current evidence is limited mainly to in vitro assays against reference strains, with little information on mechanism, stability, safety, or real‐world applicability. Future studies should therefore prioritize standardized extract characterization and validation in applied biological or food systems.

### 6.3 Anti‐Diabetic Effect

Diabetes, a chronic metabolic disorder characterized by elevated blood glucose levels, has prompted significant interest in the antidiabetic effects of natural compounds, which have shown potential in regulating blood sugar levels, enhancing insulin sensitivity, and mitigating complications associated with the disease. To understand the anti‐diabetic effect, methanol extract was prepared from *P. sativa* which was found to be composed of alkaloids, flavonoids, steroids, glycosides, triterpenoids, and saponins. The methanolic fraction displayed significant concentration‐dependent inhibition of α‐amylase and α‐glucosidase where the effect was highly comparable with the standard, acarbose. The bioactivity was further verified by administering the extract to diabetic rats. The preparation at the level of 200 mg/kg reduced blood glucose level from 208.33 mg/dL to 106.38 mg/dL and the effect was more prominent when the concentration was raised to 400 mg/kg indicating strong anti‐diabetic activity of *P. sativa* (Nagaraju et al. 2021). Despite these encouraging findings, the antidiabetic evidence remains preliminary because it is based on limited preclinical data. At present, it is not possible to determine whether the observed effects are reproducible across different extracts, doses, or experimental models, nor is it clear which constituents are primarily responsible for the activity. Further mechanistic studies, standardized extract profiling, and human investigations are needed before firm conclusions can be drawn regarding the antidiabetic relevance of *P. sativa* .

### 6.4 Cardio‐Protective Effect

Cardiovascular disease, a leading cause of mortality globally, is closely linked to oxidative stress and inflammation (Collaborators 2024a, 2024b; Santos et al. 2024). Natural compounds, such as polyphenols, flavonoids, and omega‐3 fatty acids, have demonstrated cardioprotective effects by modulating pathways related to oxidative stress, reducing inflammatory markers, improving endothelial function, and enhancing lipid profiles (Komici et al. 2020). Compounds found in *P. sativa* , such as furanocoumarins and essential oils, have shown potential in these cardioprotective roles, contributing to vascular health and offering a promising natural approach to preventing the progression of cardiovascular conditions (Zuzarte et al. 2023). To explore cardioprotective properties, a tablet form of a thick extract of the herb was prepared and subjected to rats with adrenaline‐induced myocardial dystrophy. Results showed that the preparation at the doses of 100 and 200 mg/kg contributed to a decrease in endogenous intoxication and serum urea content. Besides, the fraction restored content of reduced glutathione and catalase activity in the heart homogenate and reduced level of TBA‐active products. There was a tendency for a decrease in heart rate and an improvement in atrial conduction indicating the ability of the formulation to prevent metabolic and functional disorders of cardiac activity in conditions of adrenaline myocardial dystrophy (N. Symonenko et al. 2022; N. A. Symonenko et al. 2021). The same group further investigated the anti‐cytolytic activity against catecholamine mycocardial dystrophy in rats using the above‐mentioned parsnip herb water‐soluble thick extract to male rats for a week. The treatment decreased aspartate aminotransferase and increased reduced glutathione levels. Further, the preparation at the level of 200 mg/kg showed a pronounced antioxidant effect on the lipid peroxidation/antioxidant protection system (N. Symonenko et al. 2022; N. A. Symonenko et al. 2021). The cardioprotective evidence is currently limited and should be interpreted cautiously. The available studies are restricted to a small number of animal experiments using related extract preparations, which limits generalizability and translational relevance. Moreover, the active constituents have not been clearly linked to the observed effects, and independent validation is still lacking. Future research should focus on standardized preparations, mechanistic cardiovascular endpoints, and safety evaluation to better define the therapeutic potential of *P. sativa* .

### 6.5 Other Biological Activities

To understand the cytotoxic effect of *P. sativa* , ethanol extract was prepared using fruits and treated against several cell lines namely ML‐1 (human acute myeloblastic leukemia), J‐45.01 (human acute T cell leukemia), EOL (human eosinophilic leukemia), HL‐60 (human Caucasian promyelocytic leukemia), 1301 (human T cell leukemia lymphoblast), H‐9 (human T cell), U‐266B1 (human myeloma cells), WICL (human Caucasian normal B cell), and C‐8166 (human T cell leukemia). The EC_50_ values ranged from 34 to 300 μg/mL, where the fraction exhibited a strong effect against the WICL cell line. Overall, the fraction portrayed better activity than that of *Heracleum sibiricum* (EC_50_ 63–300 μg/mL) and *Foeniculum vulgare* (EC_50_ 122–300 μg/mL) (Bogucka‐Kocka et al. 2008). However, these cytotoxicity results remain exploratory because they are limited to in vitro cell‐line assays and do not yet establish selectivity, mechanism of action, or in vivo relevance.

Clinical evidence specifically evaluating *P. sativa* extracts or preparations in humans remains very limited, and the current pharmacological literature is dominated by in vitro and animal studies. Among the major bioactive constituents associated with the species, the best‐documented clinical evidence relates to methoxsalen (8‐methoxypsoralen), a furanocoumarin used in combination with ultraviolet A (UVA) therapy for dermatological conditions such as psoriasis. Historic clinical studies reported therapeutic efficacy of oral methoxsalen plus UVA in patients with psoriasis, and this use is also reflected in authoritative clinical monographs. Nevertheless, these clinical data cannot be directly extrapolated to *P. sativa* as a whole plant or food matrix, because the concentration, formulation, exposure conditions, and safety profile of isolated psoralens differ substantially from those of dietary or botanical preparations. Therefore, well‐designed human studies are still needed to clarify the clinical relevance of *P. sativa* and its key phytochemicals in nutrition and health contexts.

Overall, the pharmacological literature on *P. sativa* is promising but fragmented. The major research gaps include the lack of standardized extracts, insufficient comparison among subspecies and plant parts, poor alignment between phytochemical composition and biological activity, limited mechanistic work, scarce toxicological characterization, and the near absence of human evidence. These gaps currently constrain translational interpretation and should be addressed before *P. sativa* can be positioned confidently as a therapeutic or evidence‐based functional ingredient.

## 7 Functional Food Potential and Food–Nutrition Applications of *Pastinaca sativa*

**FIGURE 5:**
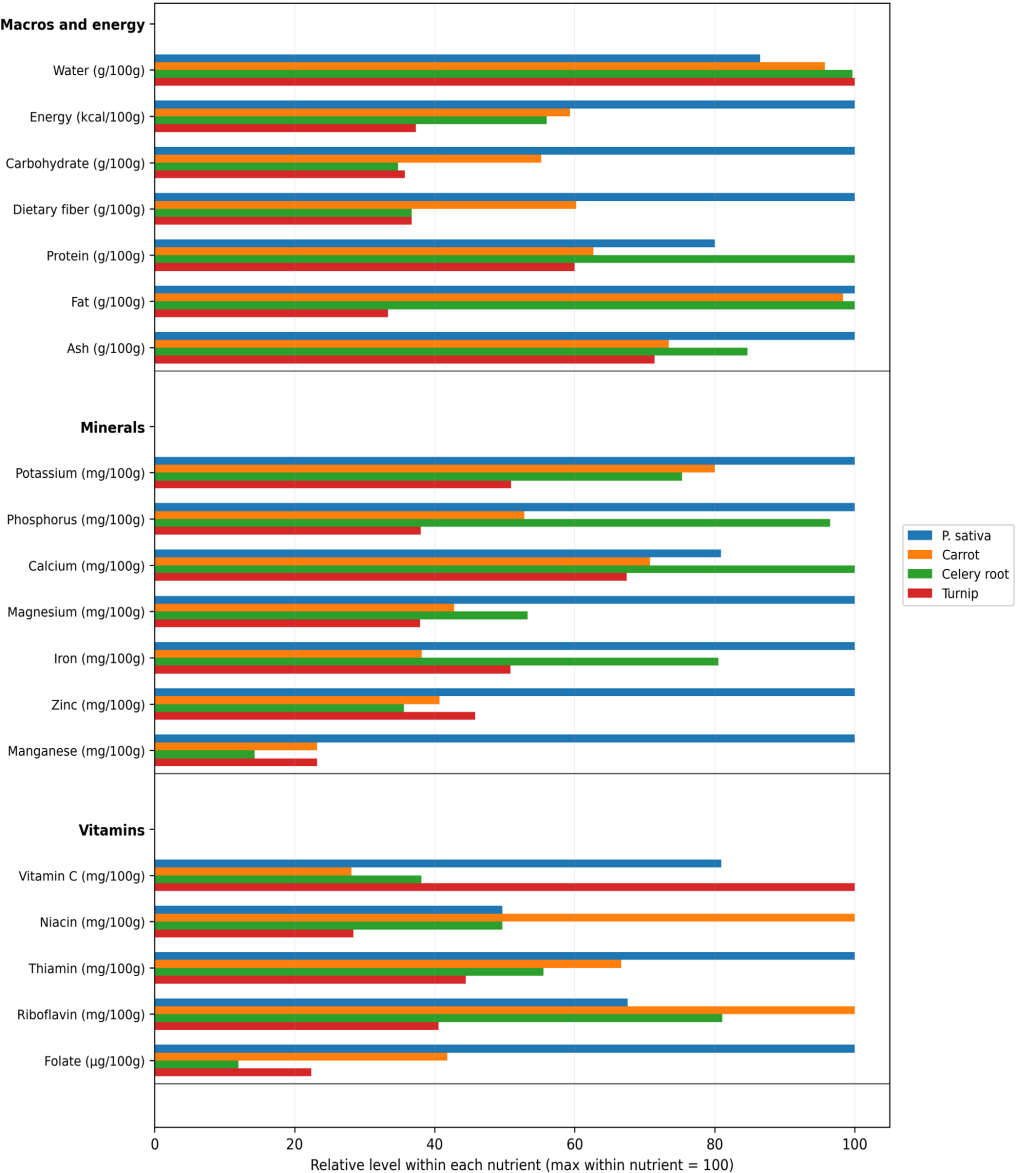
Comparative nutrient profiles of parsnip, carrot, celery root, and turnip (per 100 g, raw; row‐wise normalized).

Within this vegetable resides a multitude of vital nutrients fundamental for human well‐being, including essential minerals, dietary fiber, and an assortment of vitamins. The taproot, in particular, boasts substantial quantities of potassium, manganese, magnesium, phosphorus, zinc, and iron. Additionally, an array of noteworthy vitamins such as B1, B2, C, E, and K, alongside dietary fiber and certain protein compounds, have been documented. Notably, this vegetable is abundant in folate, also known as vitamin B9 or folic acid, a pivotal component essential for mitigating the risk of neural tube defects. *Pastinaca sativa* contains furanocoumarins such as byakangelicin (C_17_H_18_O_7_), byakangelicol (C_17_H_16_O_6_), heraclenin (C_16_H_14_O_5_), heraclenol (C_16_H_16_O_6_), isobergapten (C_12_H_8_O_4_), isobyakangelicin (C_17_H_18_O_7_), psoralen (C_11_H_6_O_3_), bergapten (C_12_H_8_O_4_), imperatorin (C_16_H_14_O_4_), isopimpinellin (C_13_H_10_O_5_), xanthotoxin (methoxsalen, C_12_H_8_O_4_), phellopterin (C_17_H_16_O_5_), and pimpinellin (C_13_H_10_O_5_). The plant possesses a spicy odor due to the presence of butyric esters in the form of heptyl, hexyl, and octyl‐butyl esters. The root is also packed with starch, carotene, vitamins, pectin, and sugars, but low in fat and sodium content. Research have shown that the root encompasses 21 g/100 g organic matter, 26.8 g/100 g dry matter (DM) dietary fiber, 1.3 g/100 g DM fructans and 5.9 g/100 g DM crude proteins resulting in low calorie content, i.e., 47 kcal/100 g. United States Department of Agriculture (USDA) database reported that raw parsnip contains quercetin of 0.99 mg/100 g (Haytowitz et al. 2018). As demonstrated in Table 5 and Figure 5, Parsnip is richer in carbohydrates and dietary fiber compared to carrot, celery, and turnip. It provides more energy and higher levels of minerals like potassium, phosphorus, magnesium, and iron. Vitamin C content is notable, although turnip is higher. Carrots surpass parsnip in niacin content. Celery is lowest in calories and macronutrients but relatively high in calcium. Overall, parsnip offers a denser nutrient profile among these vegetables (USDA). Besides, *P. sativa* root contains appreciable amount of phenolics and inulin which are known for their health beneficial effects (Tecucianu and Oancea 2019).

**TABLE 5:** Nutritional composition of *Pastinaca sativa* , carrot, celery, and turnip.

Nutrient	*P. sativa*	Carrot	Celery root	Turnip
Water (g/100 g)	79.5	87.7–88.3	88–95.2	91.9
Carbohydrate (g/100 g)	18	9.58–10.3	3.32–9.2	6.43
Dietary fiber (g/100 g)	4.9	2.8–3.1	1.8	1.8
Protein (g/100 g)	1.2	0.94	1.5	0.9
Fat (g/100 g)	0.3	0.24–0.35	0.3	0.1
Energy (kcal/100 g)	75	41–48	42	28
Ash (g/100 g)	0.98	0.72	0.83	0.7
Potassium (mg/100 g)	375	280–320	265–300	191
Phosphorus (mg/100 g)	71	35–40	22–115	27
Calcium (mg/100 g)	36	30–33	43–46	30
Magnesium (mg/100 g)	29	12.4	10.9–20	11
Iron (mg/100 g)	0.59	0.15–0.3	0.25–0.7	0.3
Zinc (mg/100 g)	0.59	0.24	0.09–0.33	0.27
Manganese (mg/100 g)	0.56	0.13	0.08	0.13
Vitamin C (mg/100 g)	17	5.9	8	21
Niacin (mg/100 g)	0.7	1.41	0.7	0.4
Thiamin (mg/100 g)	0.09	0.06	0.05	0.04
Riboflavin (mg/100 g)	0.05	0.058–0.09	0.06	0.03
Folate (μg/100 g)	67	19–37	8	15

**FIGURE 6:**
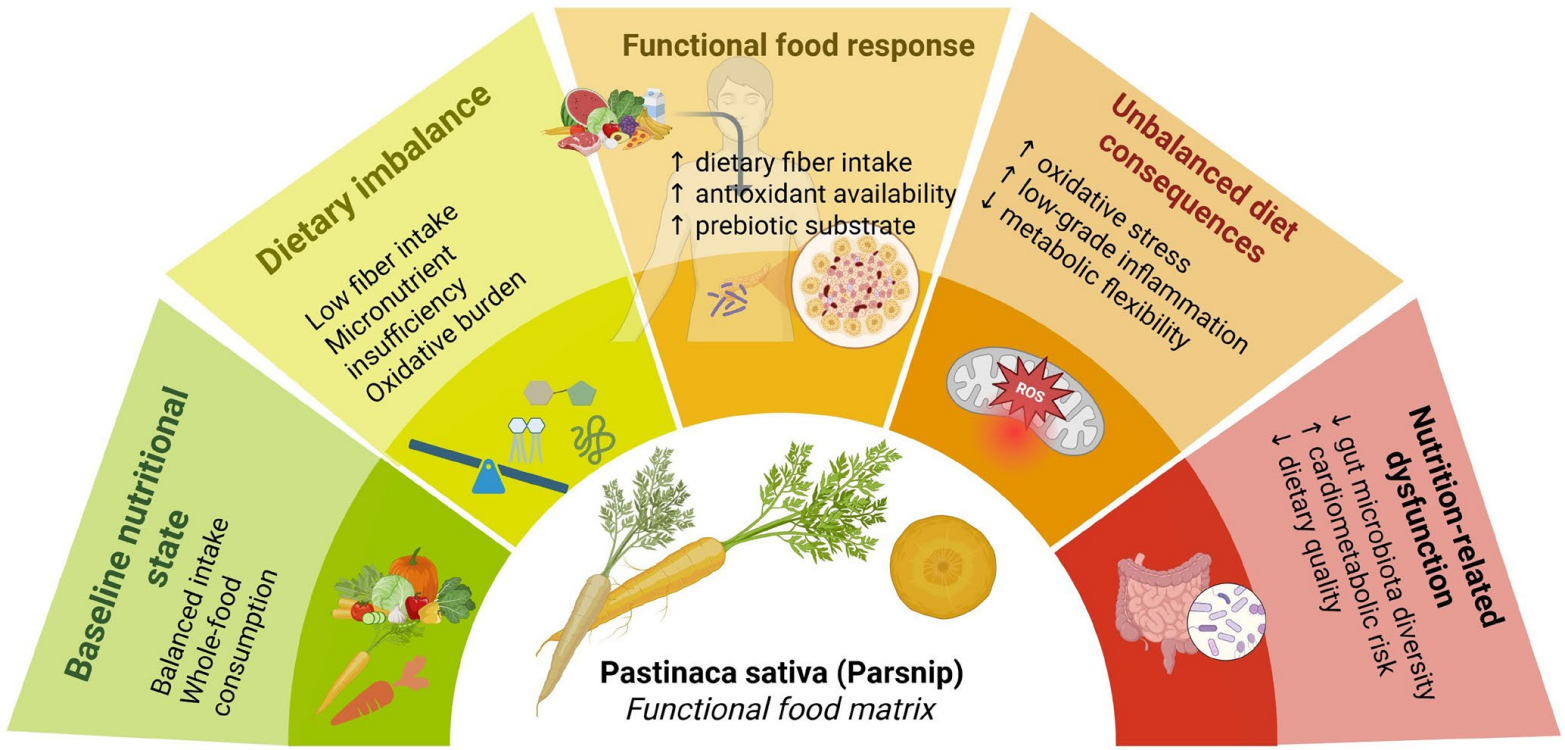
***Pastinaca sativa*** as a dietary matrix in functional food and nutrition. Schematic overview showing how fiber, prebiotic components, and antioxidant constituents of parsnip contribute to dietary balance and support nutrition‐relevant outcomes within food formulation contexts.

From an agrotechnological perspective, the quality and functional value of *P. sativa* tap roots are influenced by sowing time, planting geometry, and field management, all of which affect root size, uniformity, and marketable yield. Recent cultivation studies have shown that optimization of sowing period and cropping scheme can significantly improve tap‐root productivity under field conditions. In parallel, postharvest processing is also important for preserving or enhancing the nutritional and technological quality of parsnip roots. Recent evidence indicates that controlled postharvest aging may modify the bioactive profile and improve antioxidant‐related properties, while texture‐modification strategies such as enzymatic treatment can reduce root hardness and support the development of parsnip‐based foods for elderly and clinical nutrition. Together, these findings indicate that both cultivation practices and postharvest processing should be considered when evaluating *P. sativa* as a functional food ingredient and raw material for value‐added food applications (“Technological elements in the cultivation of parsnips ( *Pastinaca sativa* L.),” 2021). Beyond its favorable nutritional composition, *Pastinaca sativa* meets the defining criteria of a functional food by delivering physiologically active components that provide health benefits beyond basic nutrition (Arshad et al. 2025). The high dietary fiber content, particularly insoluble fiber and fructans, supports gastrointestinal function and contributes to the regulation of intestinal transit, satiety, and postprandial metabolic responses. These characteristics are directly relevant to current food–nutrition strategies aimed at improving digestive health and overall dietary quality (Arshad et al. 2025). The presence of inulin‐type fructans confers prebiotic properties, supporting the growth and metabolic activity of beneficial gut microbiota (Kalala et al. 2018). Prebiotic fibers have been consistently associated with improved gut barrier function, modulation of glucose metabolism, and support of lipid homeostasis, making parsnip‐derived ingredients suitable for functional food formulations targeting metabolic health and glycemic control (Gao et al. 2025; Lee et al. 2024). From a food application perspective, *P. sativa* demonstrates high versatility as a functional ingredient. Parsnip‐based powders, purees, and extracts can be incorporated into fiber‐enriched bakery products, vegetable‐based beverages, soups, and ready‐to‐eat meals. Its mildly sweet sensory profile allows formulation without compromising palatability, while its phenolic compounds contribute to improved oxidative stability in food systems (Lee et al. 2024) Previous food model studies have demonstrated the effectiveness of parsnip‐derived ingredients in limiting lipid oxidation, supporting their use as natural functional components in processed foods (Park et al. 2025). An additional advantage of *P. sativa* lies in its applicability to geriatric and clinical nutrition. Enzymatic processing techniques enable texture modification, improving chewability and digestibility while preserving fiber content and antioxidant capacity (Raheem et al. 2021). Such adaptations address age‐related nutritional challenges and position parsnip‐based foods as suitable components of texture‐modified diets without reliance on synthetic additives (Raheem et al. 2021). Collectively, the integration of dietary fiber, prebiotic fructans, essential minerals, and bioactive phytochemicals, combined with favorable sensory and technological properties, supports the classification of *Pastinaca sativa* as a multifunctional functional food ingredient (Kim et al. 2022). Its use extends beyond traditional culinary applications to evidence‐based food formulations aimed at enhancing dietary quality, metabolic balance, and preventive nutrition. The relationships between nutritional composition, bioactive components, food applications, and nutrition‐related outcomes are schematically illustrated in Figure 6.

## 8 Toxicity and Safety Assessment

The wild parsnip boasts a diverse collection of pro‐oxidant secondary metabolites recognized as furanocoumarins. These substances derive their toxicity from their unique capacity to capture ultraviolet light energy, forging crosslinks with DNA and engaging with ambient oxygen to yield harmful oxygen species. Within this plant, both mechanical harm and insect herbivory trigger shifts in furanocoumarin production. The synthesis of these compounds can surge by a factor of up to three, with this enhancement being specific and predominantly confined to the leaflets affected by the damage. Periodically, neighboring leaflets on the same side of the central vein may also experience induction, although those on the opposite side of the damaged leaflet remain unaffected (Berenbaum and Zangerl 1998). Furanocoumarins also have the potential to induce phytophotodermatitis in both humans and livestock. This condition manifests as patches of skin redness and blistering upon contact with the vegetable's sap and subsequent exposure to sunlight (Averill and DiTommaso 2007). Indeed, the ingestion of afflicted parsnip has been linked to phototoxic reactions. The findings presented here underscore that diseased parsnip, when present in significant concentration, can exert toxic impacts on internal tissues that are shielded from external sources of UV radiation. This includes organs such as the liver, forestomach, and esophagus (Mongeau et al. 1994). Besides furanocoumarins, wild parsnip produces several other secondary compounds which together with xanthotoxin exert synergistic toxicity against certain insects; sesquiterpenes that deter insects; and fatty acid esters toxic to some lepidopteran larvae (Khadivi et al. 2023). However, significant gaps remain in understanding the precise toxicological effects of these compounds in humans, particularly regarding chronic low‐dose exposure through diet or occupational contact. There is also a critical need for dose standardization and rigorous safety validation to ensure that potential health benefits of parsnip‐derived products can be harnessed without unintended toxic risks.

## 9 Limitations

Despite the comprehensive overview presented in this review, several limitations should be acknowledged when interpreting the food and nutrition relevance of *Pastinaca sativa* . First, much of the available evidence is derived from in vitro experiments and animal‐based studies that frequently employ concentrated extracts or isolated compounds. Although these studies provide useful insights into potential bioactivities, their direct applicability to human nutrition and regular dietary intake remains limited. Another important limitation is the considerable variability in nutritional composition and bioactive content of *P. sativa* , which is influenced by cultivar selection, geographical origin, agronomic practices, environmental conditions, and post‐harvest handling. The lack of standardized cultivation and processing parameters across studies complicates direct comparison of results and limits the establishment of consistent benchmarks for functional food applications. In addition, information regarding the bioaccessibility and bioavailability of nutrients and phytochemicals following common culinary and industrial processing methods remains insufficient. Cooking, drying, fermentation, and formulation into composite foods may significantly alter the stability and efficacy of these compounds, yet such effects are still underexplored in the current literature. From a food safety perspective, long‐term dietary exposure data are limited, particularly concerning furanocoumarin levels across different cultivars and processed products. Comprehensive evaluations based on realistic consumption patterns are required to define safe intake ranges within diversified diets. Finally, relatively few studies have systematically investigated the incorporation of *P. sativa* into functional food products, including its effects on sensory quality, technological performance, consumer acceptance, and nutritional outcomes. Future research should prioritize food‐based intervention studies, standardized processing approaches, and human dietary investigations to strengthen the evidence base supporting the functional food potential of *P. sativa* .

## 10 Conclusion and Future Prospects

*Pastinaca sativa* is a nutritionally rich root vegetable with a long history of dietary use and growing scientific interest. The available evidence indicates that parsnip is a valuable source of dietary fiber, essential minerals, vitamins, and naturally occurring bioactive compounds that collectively support its relevance in food and nutrition science. When consumed as part of a balanced diet, these components may contribute to digestive health, antioxidant intake, and overall dietary quality. Beyond its traditional culinary uses, *P. sativa* shows promising potential as a functional food ingredient. Its prebiotic fiber content, antioxidant properties, and technological versatility support its incorporation into a wide range of food products, including fiber‐enriched foods, clean‐label formulations, and products designed for specific population groups such as the elderly. Importantly, when cultivated and processed appropriately, parsnip can be safely consumed at dietary levels, further supporting its suitability for food‐based applications. Looking forward, future research should focus on food‐oriented investigations that address processing effects, nutrient bioaccessibility, sensory attributes, and consumer acceptance. Well‐designed human nutrition studies and food‐based intervention trials will be essential to validate the health relevance of *P. sativa* within realistic dietary contexts. Such efforts will facilitate the evidence‐based integration of this traditional root vegetable into modern functional food systems and support its role in promoting nutrition and public health. Future progress in *P. sativa* research may also benefit from advances in nanotechnology, biotechnology, and agrotechnology. In the context of functional foods and phytochemical delivery, nanotechnology‐based systems may improve the stability, bioaccessibility, and targeted delivery of plant‐derived bioactive compounds, thereby increasing their translational value in food and health applications. In parallel, recent progress in Apiaceae research indicates that omics‐assisted breeding, molecular markers, and gene‐based approaches can support the identification of germplasm with desirable agronomic and phytochemical traits, which may be useful for future improvement of *P. sativa* . From an agrotechnological perspective, precision cultivation strategies and innovative input‐management approaches may further enhance crop performance, yield stability, and raw‐material quality. Although these approaches remain insufficiently explored specifically in *P. sativa* , they represent promising directions for future research aimed at strengthening its value as a functional food and phytopharmaceutical resource.

## Author Contributions

**Somanjana Khatua:** writing – original draft, writing – review and editing, methodology, investigation, data curation. **Javad Sharifi‐Rad:** writing – original draft, writing – review and editing, visualization, validation, methodology, investigation, conceptualization, project administration, supervision, data curation. **Daniela Calina:** investigation, writing – original draft, writing – review and editing, visualization, validation, methodology, data curation, supervision, project administration. **Jit Sarkar:** investigation, writing – original draft, writing – review and editing, methodology, data curation. **Surajit Sen:** investigation, writing – original draft, writing – review and editing, methodology, data curation. **William N. Setzer:** writing – review and editing, visualization, validation, methodology, data curation, supervision, investigation. **Anish Nag:** writing – original draft, writing – review and editing, methodology, investigation, data curation. **Krishnendu Acharya:** investigation, writing – original draft, writing – review and editing, visualization, validation, methodology, data curation, supervision.

## Funding

The authors have nothing to report.

## Ethics Statement

The authors have nothing to report.

## Conflicts of Interest

The authors declare no conflicts of interest.

## Data Availability

The authors have nothing to report.
